# Rapid Detection and Differentiating of the Predominant *Salmonella* Serovars in Chicken Farm by TaqMan Multiplex Real-Time PCR Assay

**DOI:** 10.3389/fcimb.2021.759965

**Published:** 2021-09-29

**Authors:** Suhua Xin, Hong Zhu, Chenglin Tao, Beibei Zhang, Lan Yao, Yaodong Zhang, Dossêh Jean Apôtre Afayibo, Tao Li, Mingxing Tian, Jingjing Qi, Chan Ding, Shengqing Yu, Shaohui Wang

**Affiliations:** Shanghai Veterinary Research Institute, Chinese Academy of Agricultural Sciences, Shanghai, China

**Keywords:** multiplex real-time PCR, chicken, detection, differentiation, *Salmonella* serovars

## Abstract

*Salmonella* has been known as an important zoonotic pathogen that can cause a variety of diseases in both animals and humans. Poultry are the main reservoir for the *Salmonella* serovars *Salmonella* Pullorum (*S*. Pullorum), *Salmonella* Gallinarum (*S*. Gallinarum), *Salmonella* Enteritidis (*S*. Enteritidis), and *Salmonella* Typhimurium (*S*. Typhimurium). The conventional serotyping methods for differentiating *Salmonella* serovars are complicated, time-consuming, laborious, and expensive; therefore, rapid and accurate molecular diagnostic methods are needed for effective detection and prevention of contamination. This study developed and evaluated a TaqMan multiplex real-time PCR assay for simultaneous detection and differentiation of the *S*. Pullorum, *S*. Gallinarum, *S*. Enteritidis, and *S.* Typhimurium. In results, the optimized multiplex real-time PCR assay was highly specific and reliable for all four target genes. The analytical sensitivity corresponded to three colony-forming units (CFUs) for these four *Salmonella* serovars, respectively. The detection limit for the multiplex real-time PCR assay in artificially contaminated samples was 500 CFU/g without enrichment, while 10 CFU/g after pre-enrichment. Moreover, the multiplex real-time PCR was applied to the poultry clinical samples, which achieved comparable results to the traditional bacteriological examination. Taken together, these results indicated that the optimized TaqMan multiplex real-time PCR assay will be a promising tool for clinical diagnostics and epidemiologic study of *Salmonella* in chicken farm and poultry products.

## Introduction

*Salmonella* is an important zoonotic pathogen that can cause a variety of diseases in both animals and humans (Majowicz et al., 2010). *Salmonella* is prevalent in domestic animals such as poultry, pigs, and cattle. Poultry are a main reservoir for *Salmonella*, including the most prevalent *Salmonella* serovars *Salmonella* Pullorum (*S*. Pullorum), *Salmonella* Gallinarum (*S*. Gallinarum), *Salmonella* Enteritidis (*S*. Enteritidis), and *Salmonella* Typhimurium (*S*. Typhimurium) (Medalla et al., 2016; Wang et al., 2020; Xu et al., 2020; Zhao et al., 2020; Yu et al., 2021). These *Salmonella* serovars can lead to serious avian salmonellosis, which causes economic losses in the poultry industry. In addition, *Salmonella* can be transmitted to humans by contaminated poultry products and cause acute gastroenteritis or diarrhea, being a threat to public health (Balasubramanian et al., 2019). Currently, animals, in particular poultry, are considered to be the primary cause for salmonellosis and numerous other foodborne outbreaks (Keerthirathne et al., 2017; Biswas et al., 2019; Liu et al., 2021). Thus, detection and differentiation of these *Salmonella* serovars in poultry farms are required to prevent, control, and eliminate the spread of *Salmonella*.

Rapid and accurate diagnosis is curial for effective prevention and control of the disease. Currently, more than 2,600 *Salmonella* serovars have been identified based on the O, H, and Vi antigens (Issenhuth-Jeanjean et al., 2014). Although bacteriological culture and serum agglutination test were considered to be the gold standard for differentiating *Salmonella* serovars, there were many disadvantages for this routine diagnosis in practice. The conventional culture method tends to be complex, time-consuming, and laborious. Moreover, false-negative result for O and H antigens agglutination test occurs occasionally due to the loss of surface antigens in non-culturable state (Schrader et al., 2008). In efforts to avoid such disadvantages, several nucleic acid amplification methods have been developed to detect and differentiate the *Salmonella* serovars (Shi et al., 2015). Although there is extensive sequence conservation in *Salmonella* genome, comparative genomic analysis is effective to validate novel serovar-specific genes. The unique genes had been identified among the different *Salmonella* serovars (Liu et al., 2011; Zhang et al., 2019). The gene *lygD* in Sdf locus has been found specific in *S*. Enteritidis. Serovar-specific gene *STM4495* for identifying *S*. Typhimurium was obtained by comparative genomics (Agron et al., 2001; Akiba et al., 2011). In addition, comparative analysis of the *glgC* gene sequence identified an 11 bp (GATCGATCACG) deletion presented only in *S.* Gallinarum but not other *Salmonella* serovars (Kang et al., 2011). Based on these specific gene, PCR assays were applied for detecting different *Salmonella* serovars (Shah et al., 2005; Kim et al., 2006; Hong et al., 2008; Kang et al., 2011; Xu et al., 2018). Compared to conventional PCR, real-time PCR assay offers advantages in rapidity, quantitative measurement, and avoiding of cross-contamination. More importantly, real-time PCR assay enables to obtain both qualitative and quantitative measurement of the pathogen presented in samples. Thus, real-time PCR has been widely utilized to detect different pathogens (Ding et al., 2017; Liu et al., 2019). Recently, increasing studies developed single and multiplex real-time PCR for the specific detection of major *Salmonella* serovars in food products (Lee et al., 2009; Munoz et al., 2010; Prendergast et al., 2013; Kasturi and Drgon, 2017; Kim et al., 2017; Nair et al., 2019). The rapid detection and differentiation of *Salmonella* serovars are required for the epidemiologic investigation of *Salmonella* in chicken farms.

This study attempted to develop a rapid multiplex RT-PCR assay for the simultaneous detection and differentiation of the prevalent *S*. Pullorum, *S*. Gallinarum, *S*. Enteritidis, and *S.* Typhimurium. The specificity and sensitivity evaluations indicated that the developed multiplex real-time PCR assay appears to be a promising tool for clinical diagnostics and epidemiology studies for *Salmonella* in chicken farm and poultry products.

## Materials and Methods

### Bacterial Strains and Growth Conditions

The *Salmonella* and non-*Salmonella* bacterial strains used to establish and verify the multiplex real-time PCR assay are listed in **Table 1**. The *Salmonella*, *Escherichia coli*, and *Pseudomonas aeruginosa* strains were cultured at 37°C in Luria-Bertani (LB) medium (BD, Detroit, MI, USA) with aeration. Other bacterial strains were grown in appropriate medium under recommended culture conditions.

**Table 1:** Specificity of the multiplex real-time PCR for different bacterial strains.

Bacteria	No. of strains	Multiplex real-time PCR results			
		*S.* Pullorum	*S.* Gallinarum	*S.* Enteritidis	*S.* Typhimurium
***Salmonella* strains**					
*S.* Pullorum CVCC519	1	1	0	0	0
*S.* Gallinarum ATCC19945	1	0	1	0	0
*S.* Typhimurium 14028	1	0	0	0	1
*S.* Enteritidis CVCC1805	1	0	0	1	0
*S.* Typhimurium SL1344	1	0	0	0	1
*S.* Pullorum isolates	36	36	0	0	0
*S.* Gallinarum isolates	5	0	5	0	0
*S.* Typhimurium isolates	50	0	0	0	50
*S.* Enteritidis isolates	35	0	0	35	0
*S.* Anatum CAU0118	1	0	0	0	0
*S.* Agona BNCC192235	1	0	0	0	0
S. Anatis CMCC50774	1	0	0	0	0
*S.* Choleraesuis CVCC503	1	0	0	0	0
*S.* Newbort ATCC6962	1	0	0	0	0
*S.* Dublin CMCC50042	1	0	0	0	0
*S.* Heidelberg CMCC50111	1	0	0	0	0
*S.* Paratyphi B CMCC50094	1	0	0	0	0
*S.* Derby CMCC50112	1	0	0	0	0
*S.* Derby isolates	5	0	0	0	0
**Non-*Salmonella* strains**					
*E. coli* O157:H7 ATCC35150	1	0	0	0	0
*E. coli* O157:H7 ATCC43889	1	0	0	0	0
*E. coli* O38 CVCC1543	1	0	0	0	0
*E. coli* O73 CVCC1547	1	0	0	0	0
*E. coli* O78 CGMCC10602	1	0	0	0	0
*E. coli* isolates	50	0	0	0	0
*Klebsiella pneumoniae* isolates	10	0	0	0	0
*Listeria monocytogenes* 10403s	1	0	0	0	0
*Listeria monocytogenes* EGD	1	0	0	0	0
*Listeria monocytogenes* isolates	20	0	0	0	0
*Pasteurella multocida* isolates	10	0	0	0	0
*Riemerella anatipestifer* isolates	20	0	0	0	0
*Staphylococcus aureus* ATCC25923	1	0	0	0	0
*Staphylococcus aureus* isolates	15	0	0	0	0
*Pseudomonas aeruginosa* PAO1	1	0	0	0	0
*Pseudomonas aeruginosa* isolates	10	0	0	0	0
*Proteus mirabilis* isolates	6	0	0	0	0

### DNA Extraction

Bacterial genomic DNA was extracted from fresh bacterial culture using TIANamp Bacteria DNA Kit (Tiangen Biotech, Beijing, China) following the manufacturer’s instructions. Whereas, the total DNA from clinical samples was prepared using DNA Isolation Reagent for meat Products (Tiangen Biotech, Beijing, China). The concentration and purity of the DNA were measured with spectrophotometer.

### Primers and Probes Designing

**Figure 1:**
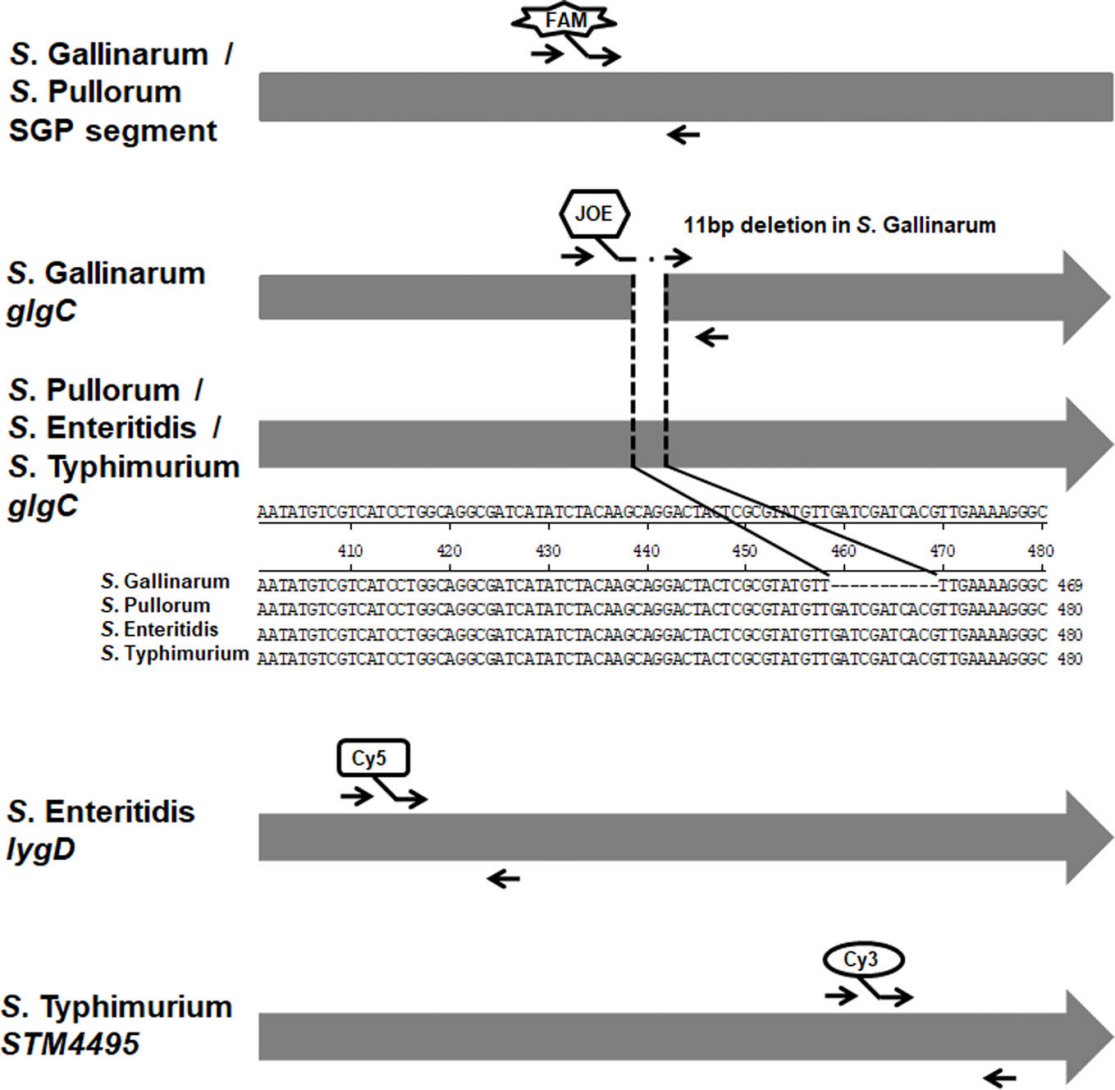
Diagram of the primers and probes designing for the multiplex real-time PCR. The specific gene or segment of these four *Salmonella* serovars was analyzed and exploited to design the primers and probes. The primers and fluorophore-labeled probes were indicated. The arrows indicated the positions of the designed primers. In addition, the alignments of *glgC* genes in *S*. Gallinarum, *S*. Pullorum, *S*. Enteritidis, and *S.* Typhimurium were shown.

To design the suitable primers, the specific gene sequences of these *Salmonella* serovars were analyzed. By bioinformatics analysis, we found a specific gene segment (699 bp) SGP (GenBank No. CP012347.1 segment 2766328 to 2767027) was presented and generally conserved in *S.* Pullorum and *S.* Gallinarum. In addition, a previous study identified an 11 bp (GATCGATCACG) sequence deletion in *glgC* gene presented only in *S.* Gallinarum, but not other *Salmonella* serovars (Kang et al., 2011). Thus, the SGP gene segment and truncated sequence of *glgC* gene could be exploited to differentiate *S.* Pullorum and *S.* Gallinarum from other *Salmonella* serovars. The serovar-specific genes *lygD* and *STM4495* for specifically identifying and differentiating *S*. Enteritidis and *S*. Typhimurium were selected as targets according to previous studies (Agron et al., 2001; Akiba et al., 2011). Then, the specific primers and probes were designed based on the specific gene sequences (**Table 2** and **Figure 1**). Furthermore, the specificity of the primer sequences was tested by *in silico* analysis using BLAST at the National Center for Biotechnology Information (NCBI). All the primers and probes were synthesized by Sangon Biotech (Shanghai) Co., Ltd, China.

**Table 2:** Primers and probes used for the multiplex real-time PCR.

Primers or probes	Sequence (5’ to 3’)	Optimal concentration	Target serovars/gene segments	*Salmonella* serovars			
				*S.* Pullorum	*S.* Gallinarum	*S.* Enteritidis	*S.* Typhimurium
SGP-F	GGATGTCCACGCTCATTTCTC	0.05 μM	*S.* Pullorum and *S.* Gallinarum/*SGP* (CP012347.1, 2766328-2767027)	+	+	-	-
SGP-R	TGAAAGCTGGCGTTACGGTTA	0.05 μM					
SGP- Probe	FAM-CGTCAGGCCCACCGCCGACAG-BHQ1	0.05 μM					
SG-F	CAGGCGATCATATCTACAAGCAGG	0.1 μM	*S.* Pullorum and *S.* Gallinarum*/glgC* (11 bp deletion in *S*. Pullorum)	-	+	-	-
SG-R	TCTTGTCGCTTTCATCGACCGC	0.1 μM					
SG- Probe	JOE-ACTCGCGTATGTTTTGAAAAGGGC-BHQ1	0.05 μM					
SE-F	TCTGGGACGCCAAAAAGC	0.1 μM	*S.* Enteritidis/*lygD*	-	-	+	-
SE -R	TGACGGTAGATTGTGTCTCAAAGC	0.1 μM					
SE- Probe	Cy5-TCAAACTTACTCAGGAGATCGCCGCTG-BHQ2	0.05 μM					
ST-F	GTTCAGCTCCGGTAAAGAGAA	0.2 μM	*S.* Typhimurium/*STM4495*	-	-	-	+
ST-R	AGCAGCGGCACTACATATTC	0.2 μM					
ST-Probe	Cy3-CGTTTGAGTGCCTGGTCTATCTGA-BHQ2	0.4 μM					

### Optimization and Development of Multiplex Real-Time PCR Assay

The multiplex real-time PCR was carried out in a final volume of 20.0 µl, and the concentrations of primers, probes, and reaction condition were optimized using the purified DNA of *S.* Pullorum, *S.* Gallinarum, *S.* Enteritidis, and *S.* Typhimurium reference strains (**Table 1**). Sterile distilled water was used as negative control. The multiplex real-time PCR was performed on the ABI 7500 Real-time PCR system (Applied Biosystems, CA, USA), and fluorescent signals were detected simultaneously during annealing/extension phase. All analyses were performed with ABI 7500 Software Version 1.4 (Applied Biosystems, CA, USA).

### Specificity of the Multiplex Real-Time PCR Assay

A collection of bacterial strains, including various *Salmonella* serovars and non-*Salmonella* (**Table 1**), was used to evaluate the specificity of multiplex real-time PCR assay. All of the bacterial strains have been confirmed by biochemical identification, PCR, and serotyping with traditional agglutination assay. The bacterial genomic DNA was extracted and used as a template in the multiplex real-time PCR assay under optimized condition.

### Standard Curve and Sensitivity Analysis

The standard curves and sensitivity of multiplex real-time PCR were determined using genomic DNA of various bacterial concentrations as described previously (Lee et al., 2009; Ding et al., 2017). The pure cultures of the *S.* Pullorum, *S.* Gallinarum, *S.* Enteritidis, and *S.* Typhimurium strains were 10-fold serially diluted to appropriate dilutions (ranging from 3 to 3×10^7^ CFU/ml), which were counted by plating. The genomic DNA extracted from bacterial culture dilutions was used as templates for multiplex real-time PCR. Negative control includes sterile distilled water in place of DNA. The standard curves were calculated automatically based on the Cycle threshold (Ct) values using the ABI 7500 Software. The amplification efficiencies (E) were determined by using the slope of the standard curve and applying the equation: E = (10^−1/slope^)-1 (Kawasaki et al., 2010).

### Evaluation of the Limit of Detection of Multiplex Real-Time PCR in Artificial Contamination Samples

The LOD of multiplex real-time PCR assay was evaluated for the artificial contamination samples with or without enrichment as previously described (Lee et al., 2009), with some modifications. Briefly, 100 μl of each bacterial dilutions (1 to 10^8^ CFU/ml) were individually added to 1 g of chicken meat samples. Then, these contaminated samples were thoroughly homogenized with 9 ml of buffered peptone water (BPW). The pre-enriched homogenized samples were used for DNA extraction using DNA Isolation Reagent for meat Products (Tiangen Biotech, Beijing, China). In addition, the homogenized samples were incubated at 37°C for 6 h. After primary enrichment, DNA extraction was performed. These DNA were used as templates for multiplex real-time PCR. Non-inoculated meat was subjected to the same procedure and used as a negative control.

### Detection of Clinical Samples

The multiplex real-time PCR assay was applied to evaluate the presence of *Salmonella* in 60 sick or dead chicken with clinical signs collected from five farms. All animal experiments were conducted in strict accordance with the guidelines of the Humane Treatment of Laboratory Animals and were approved by the Animal Care and Use Committee at the Shanghai Veterinary Research Institute, China. The liver samples were collected aseptically from the chickens. The samples were homogenized for primary enrichment or DNA isolation as described above. The DNA extracted from these samples was analyzed by multiplex real-time PCR method. Meanwhile, each sample was also subjected to standard bacterial culture methods and traditional serum agglutination assay.

## Results

### Development of the Multiplex Real-Time PCR Assay

Based on the bioinformatics analysis, the specific primers and probes were designed based on the target genes of these four *Salmonella* serovars (**Figure 1**). Then, multiplex real-time PCR was optimized by adjustment of different parameters. The optimal amplification reaction contained 10.0 µl Premix Ex Taq™ (Takara, Dalian, China), 0.2 μl ROX Reference Dye II (Takara, Dalian, China), each primer and probe at final concentrations of 0.05 to 0.4 μM (**Table 2**), 2.0 μl template and nuclease-free water. The reaction profile consisting of initial denaturation at 95°C for 30 s, followed by 40 cycles of denaturation at 95°C for 5 s and 40 s annealing/extension at 57°C. Accordingly, the reference strains of these four *Salmonella* serovars were specifically differentiated by the multiplex real-time PCR assay.

### Analytical Specificity

**Figure 2:**
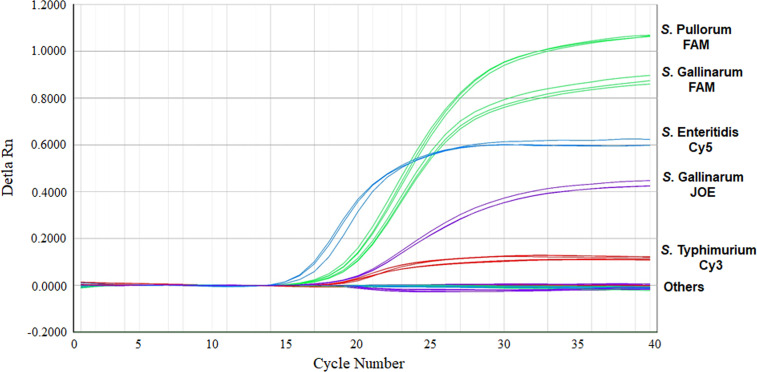
Specificity of the multiplex real-time PCR for the detection and differentiation of *S*. Pullorum, *S*. Gallinarum, *S*. Enteritidis, and *S.* Typhimurium. Others: None of these four *Salmonella* serovars bacteria.

The specificity of the multiplex real-time PCR was evaluated using the different bacterial templates listed in **Table 1**. All the *S.* Pullorum, *S.* Gallinarum, *S.* Enteritidis, and *S.* Typhimurium strains produced the corresponding amplified signals (**Figure 2**). Whereas, the non-target bacteria, including other *Salmonella* serovars and non-*Salmonella* strains, yielded negative results in the multiplex real-time PCR (**Table 1**). No false positive or negative results were found, indicating the multiplex real-time PCR was specific.

### Standard Curve and Sensitivity of the Multiplex Real-Time PCR Assay

**Figure 3:**
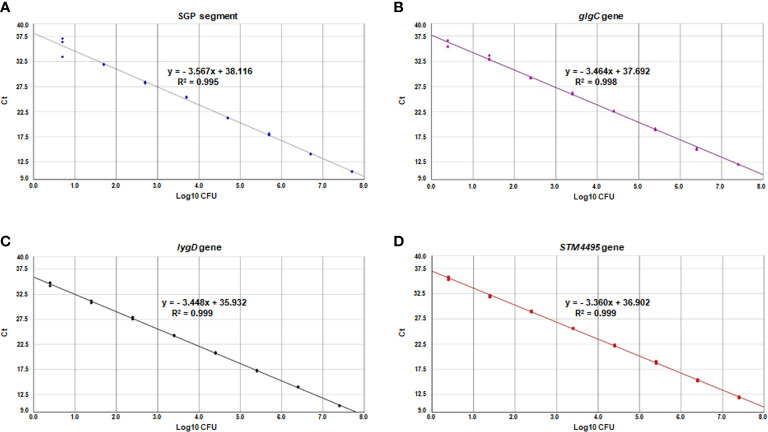
Standard curves of the multiplex real-time PCR for SGP segment **(A)**, *glgC* gene **(B)**, *lygD* gene **(C)**, and *STM4495* gene **(D)** using serially diluted *S*. Pullorum **(A)**, *S*. Gallinarum **(B)**, *S*. Enteritidis **(C)**, and *S.* Typhimurium **(D)** bacterial DNA, respectively.

The standard curve of the multiplex real-time PCR assay was constructed using the mean Ct values for various *Salmonella* concentrations corresponding to the genomic DNA. As shown in **Figure 3**, the slopes of the standard curves for *S.* Pullorum, *S.* Gallinarum, *S.* Enteritidis, *S.* Typhimurium were −3.567, −3.464, −3.448, and −3.360, respectively. The correlation coefficients (R^2^) were above 0.99, and the amplification efficiencies ranged from 90 to 110%, indicating high linearity for the multiplex real-time PCR assay. The sensitivity analysis showed that the bacterial DNA corresponding to 3 CFU of these four *Salmonella* serovars could be detected for the multiplex real-time PCR assay.

### Limit of Detection in Artificially Contaminated Chicken Samples

The artificially contaminated samples with serial dilutions of each *Salmonella* were tested for the LOD of multiplex real-time PCR assay. For the samples without enrichment, each *Salmonella* of 500 CFU/g could be detected by the multiplex real-time PCR assay. However, when incubated for 6 h for the enrichment, the multiplex real-time PCR assay could successfully detect as low as 10 CFU of each *Salmonella* in 1 g chicken samples.

### Clinical Samples Validation

To evaluate the discernibility and applicability of established method for clinical samples, a total of 60 suspected samples were collected and detected using our multiplex real-time PCR and conventional bacteriological tests. After enrichment, 35 of the 60 clinical samples were *Salmonella* positive by multiplex real-time PCR, whereas other samples had no *Salmonella*. Among these positive samples, 21 samples were identified as *S.* Pullorum, one for *S.* Gallinarum, nine for *S.* Enteritidis, and four for *S.* Typhimurium. Same samples were also examined by traditional bacteriological serotyping tests, which was in accordance with the multiplex real-time PCR results with enrichment. Whereas, two *Salmonella* positive samples gave negative results without the additional enrichment step (**Table 3**). This might be due to the limited bacterial amounts in the samples. Thus, the results indicated that the multiplex real-time PCR assay with enrichment is more sensitive than pre-enriched condition to detect the limited amounts of bacteria in samples.

**Table 3:** Consistency evaluation of the multiplex real-time PCR and conventional bacteriological method for the clinical samples.

	*S*. Pullorum	*S*. Gallinarum	*S*. Enteritidis	*S*. Typhimurium	Total
Multiplex real-time PCR with enrichment	21	1	9	4	35
Multiplex real-time PCR without enrichment	20	1	8	4	33
Bacteriological	21	1	9	4	35

## Discussion

It has been shown that *S*. Pullorum, *S*. Gallinarum, *S*. Enteritidis, and *S.* Typhimurium were the prevalent pathogens of salmonellosis in chicken farms (Medalla et al., 2016; Wang et al., 2020; Xu et al., 2020; Zhao et al., 2020; Yu et al., 2021). In addition, *S*. Enteritidis and *S*. Typhimurium could lead to serious zoonotic diseases *via* contaminated food, including poultry products (Coburn et al., 2007). Conventional, *Salmonella* serovars were identified according to the Kauffman-White scheme based on the specific cell-surface O and H antigens. Although serum agglutination assay offers a reliable method for differentiating *Salmonella* serovars, it is labor-intensive, complex, costly, and time-consuming (Schrader et al., 2008). Nowadays, reducing cost and time of experiment are critical for pathogen detection. Therefore, the rapid and accurate detection method has the potential to be of great significance for preventing the spread of salmonellosis. Several molecular methods, such as PCR and loop-mediated isothermal amplification, exist for identifying various *Salmonella* serovars with advantages in sensitivity, specificity, and speed (Shi et al., 2015; Yang et al., 2018). Among them, real-time PCR is more sensitive and suitable for high-throughput analysis (Kralik and Ricchi, 2017). Thus, this study developed a multiplex real-time PCR assay that simultaneously detected and differentiated the prevalent *S*. Pullorum, *S*. Gallinarum, *S*. Enteritidis, and *S.* Typhimurium, which exhibited efficiently identification in cultured bacteria and chicken samples.

Selection of specific target genes and design of compatible primers and probes are critical for the proper detection specificity of nucleic acid amplification. Although the homology of the genomes of various *Salmonella* serovars was very high, some genes were found to be related to specific serovars. Various genes, such as genes encoding the O, H, and Vi antigens (*rfb*, *fliC*, *fliB*, *viaB*, *ipaJ*), have been candidates suitable for the specific detection and serotyping of *Salmonella* in diverse clinical samples (Hirose et al., 2002; Hong et al., 2008; Xu et al., 2018). In the present study, analysis of genomic sequences identified a gene segment SGP (GenBank No. CP012347.1 segment 2766328 to 2767027) specifically existing in all *S*. Pullorum and *S*. Gallinarum. A previous study has revealed that the *glgC* gene is deemed to be the preferred target for differentiating *S*. Pullorum and *S*. Gallinarum from other *Salmonella* serovars (Kang et al., 2011). Based on literature (Agron et al., 2001; Akiba et al., 2011), the *S*. Enteritidis specific *lygD* gene and *STM4495* gene specific for *S*. Typhimurium were chosen as targets in this study. As a result, the primers and probes were designed and optimized targeting these specific genes. Furthermore, no mismatch in the primers and probes with the available bacterial genome in GenBank was found by *in silico* analysis. The developed multiplex real-time PCR showed excellent specificity and exclusivity by the detection of *Salmonella* strains as well as other bacterial species. No cross-reactivity, false positives, or false negatives were observed. Previously, real-time PCR assays targeting various specific genes had been applied for *Salmonella* (Lee et al., 2009; Kasturi and Drgon, 2017; Kim et al., 2017; Nair et al., 2019). This study incorporated these four target genes into a unified multiplex real-time PCR assay for the detection and differentiation of multiple *Salmonella* serovars in chicken samples.

In the analytical sensitivity evaluation, the developed multiplex real-time PCR assay was shown to detect as low as the bacterial DNA corresponding to 3 CFU/ml bacterial cultures. Although the LOD of this multiplex real-time PCR was 500 CFU/g in artificially contaminated chicken samples without enrichment, it had more improved detection limits and yielded positive results even at the lowest contamination levels tested (10 CFU/g chicken samples) for the enriched samples. There was some uncertainty, such as PCR inhibitors, competitor organisms, which might result in the lower LOD without enrichment step. Indeed, the pre-enrichment step was effective to increase the number of bacterial cells and to dilute inhibitory substances that exist in the sample. Thus, an additional enrichment step was actually applied to increase the sensitivity of the multiplex real-time PCR (Lee et al., 2009; Ding et al., 2017). The LOD of this multiplex real-time PCR assay was similar to previous studies (Munoz et al., 2010; Kasturi and Drgon, 2017; Liu et al., 2019), indicating its sensitivity for diagnostic purpose. Our multiplex real-time PCR was applied to detect these four *Salmonella* serovars in poultry clinical samples, which achieved same results comparable to the traditional bacteriological examination. However, two of the positive samples were tested as negative by multiplex real-time PCR lack of enrichment step. The possibility remains the amplification inhibition factors or low bacterial concentration in the samples (Lee et al., 2009; Ding et al., 2017). In terms of shortening time for multiple bacterial detection, the entire process of the multiplex real-time PCR assay from sample enrichment to data analysis can be completed in 12 h. The effectiveness of multiplex real-time PCR was significantly improved compared to the traditional culture method.

In summary, this study developed a TaqMan multiplex real-time PCR assay for simultaneous detection and differentiation of prevalent *S*. Pullorum, *S*. Gallinarum, *S*. Enteritidis, and *S.* Typhimurium. Considering the specificity, sensitivity, and effectiveness, the multiplex real-time PCR assay developed herein appears to be a promising tool for clinical diagnostics and epidemiologic study of *Salmonella* in chicken farm and poultry products.

## Data Availability Statement

The original contributions presented in the study are included in the article/supplementary material. Further inquiries can be directed to the corresponding authors.

## Ethics Statement

The animal study was reviewed and approved by the Animal Care and Use Committee at the Shanghai Veterinary Research Institute, China.

## Author Contributions

SW conceived the project. SX and HZ developed the TaqMan multiplex real-time PCR assay and wrote the original manuscript draft. CT, BZ, LY, YZ, and DA were responsible for sampling and sample test. TL, MT, JQ, CD, SY, and SW analyzed and discussed the experimental results. SW and SY directed the experiments, funded the research, and edited the manuscript. All authors contributed to the article and approved the submitted version.

## Funding

This work was supported by the National Key Research and Development Program of China (2016YFD0500800, 2018YFD0500500), the National Natural Science Foundation of China (31972654), Shanghai Pujiang Program (2019PJD057), Scientific and Technical Innovation Project of the Chinese Academy of Agricultural Sciences, China (SHVRI-ASTIP-2014-8), and the National Basic Fund for Research Institutes, which is supported by the Shanghai Veterinary Research Institute, Chinese Academy of Agricultural Sciences (2021JB07).

## Conflict of Interest

The authors declare that the research was conducted in the absence of any commercial or financial relationships that could be construed as a potential conflict of interest.

## Publisher’s Note

All claims expressed in this article are solely those of the authors and do not necessarily represent those of their affiliated organizations, or those of the publisher, the editors and the reviewers. Any product that may be evaluated in this article, or claim that may be made by its manufacturer, is not guaranteed or endorsed by the publisher.
